# Effects of a Novel Bioactive Glass Composition on Biological Properties of Human Dental Pulp Stem Cells

**DOI:** 10.3390/ma13184049

**Published:** 2020-09-12

**Authors:** Rosanna Di Tinco, Rachele Sergi, Giulia Bertani, Alessandra Pisciotta, Devis Bellucci, Gianluca Carnevale, Valeria Cannillo, Laura Bertoni

**Affiliations:** 1 Department of Surgery, Medicine, Dentistry and Morphological Sciences with Interest in Transplant, Oncology and Regenerative Medicine, University of Modena and Reggio Emilia, Via del Pozzo 71, 41124 Modena, Italy; rosanna.ditinco@unimore.it (R.D.T.); giulia.bertani@unimore.it (G.B.); alessandra.pisciotta@unimore.it (A.P.); gianluca.carnevale@unimore.it (G.C.); 2 Department of Engineering “Enzo Ferrari”, University of Modena and Reggio Emilia, Via P. Vivarelli 10, 41125 Modena, Italy; rachele.sergi@unimore.it (R.S.); devis.bellucci@unimore.it (D.B.); valeria.cannillo@unimore.it (V.C.)

**Keywords:** bioactive glass, stem cells, human dental pulp stem cells (hDPSCs), bone regeneration, cell culture, strontium, magnesium, high crystallization temperature

## Abstract

Functional reconstruction of bone defects represents a clinical challenge in the regenerative medicine field, which targets tissue repair following traumatic injuries and disease-related bone deficiencies. In this regard, the optimal biomaterial should be safe, biocompatible and tailored in order to promote the activation of host progenitor cells towards bone repair. Bioactive glasses might be suitable biomaterials due to their composition being able to induce the host healing response and, eventually, anti-bacterial properties. In this study we investigated whether and how an innovative bioactive glass composition, called BGMS10, may affect cell adhesion, morphology, proliferation, immunomodulation and osteogenic differentiation of human dental pulp stem cells (hDPSCs). When cultured on BGMS10, hDPSCs maintained their proliferation rate and typical fibroblast-like morphology, showing the expression of stemness markers STRO-1 and c-Kit. Moreover, the expression of FasL, a key molecule in mediating immunomodulation effects of hDPSCs, was maintained. BGMS10 also proved to trigger osteogenic commitment of hDPSCs, as confirmed by the activation of bone-related transcription factors RUNX2 and Osx and the ongoing deposition of extracellular matrix supported by the expression of OPN and OCN. Our findings suggest that BGMS10 not only maintains the typical biological and immunomodulatory properties of hDPSCs but also favors the osteogenic commitment.

## 1. Introduction

Biomaterials (i.e., ceramics, glasses, metals, polymers, composites) have been widely used to repair or replace structural parts of the human body [1,2,3,4]. Biomaterials should be biocompatible, intrinsically non-toxic, non-allergenic and non-carcinogenic. Furthermore, based on their response in the host, biomaterials can be classified as first, second and third generation. First-generation biomaterials do not induce or undergo chemical or biological changes once in contact with fluids and tissues; by contrast, second-generation biomaterials can cause a reaction mechanism in biological environment which result in new bone or tissue formation [5,6]. A further step is achieved by biomaterials of the third generation, which promote a specific cellular response at molecular level [7]. Such systems are designed to gradually degrade and to be replaced by living host tissues. Among biomaterials, bioactive glasses show the capability to stimulate cells towards a path of regeneration and self-repair, being suitable for both bone and tissue reconstruction and repair. In the late 1960s, L.L. Hench developed the first bioactive glass named 45S5 [8]; since then, various bioactive glass compositions have been developed. Bioactive glasses can be classified as silicate, phosphate, and borate [8,9]. Borate-based and phosphate-based bioactive glasses are suitable for healing applications because of their high dissolution rate [10]. On the other hand, silicate-base bioactive glasses are the main category of glasses employed in clinical applications. The advantage of bioactive glasses is that their composition can be easily tuned to stimulate a specific response in the host or to introduce antibacterial properties, for example. Therefore, the main direction of investigations should be on the achievement of a better understanding of bioactive glass compositions in terms of the effects of doping elements, to further enhance the response in the host once implanted. However, the main drawback of bioactive glasses is the crystallisation upon thermal treatment, which can reduce or undermine the inherent bioactivity [8].

Recently, an innovative bioactive glass composition, called BGMS10, has been proposed to overcome the inherent limitations of the state-of-the-art materials. Such bioactive glass was designed to achieve an ultra-high crystallisation temperature and a low tendency to devitrification, being particularly suitable for applications that require heat treatment [11]. Moreover, the addition of therapeutic ions such as strontium and magnesium resulted in an improved biological response [12] also compared to 45S5 [13], as reported as well for other bioactive glasses containing Sr and Mg [14,15,16,17,18,19,20]. BGMS10 resulted as suitable for the preparation of composites [21], dental putties [22], 3D printed scaffolds [23], wound dressings [24,25], and antibacterial systems [26], being very promising also compared to other bioactive glasses investigated in literature (e.g., [27,28,29,30,31,32]).

Due to the favourable combinations of properties, BGMS10 seems to be an optimal candidate for the production of sintered products for clinical trials, such as sintered disks, scaffolds and coatings [33,34]. Before starting with costly in vivo experiments, in the present paper such bioactive glass is tested by means of in vitro test with human dental pulp stem cells (hDPSCs) as a further investigation. The choice of hDPSCs as cell source to study cells/materials interaction might be suitable due to their peculiar embryological origin from neural crest, indeed neural crest stem cells migrate towards different anatomical districts, including the tissue components of maxillofacial complex. Furthermore, hDPSCs can be isolated easily from human dental pulp tissue through routine, low-invasive tooth extraction procedures. After dental pulp digestion, the cells obtained are a heterogeneous cell population containing different cell types, among which a stem cell niche can be isolated through immune-selection against stemness markers c-Kit, the tirosin-kinase receptor of stem cells factor, and STRO-1, an antigen present in a stromal cell population containing osteogenic precursors [35,36,37]. As reported in literature, hDPSCs are characterized by a high proliferation rate, low immunogenicity and immunomodulatory properties, but they also own the capacity to differentiate into different cytotypes such as osteogenic, chondrogenic, adipogenic, myogenic and neural lineages [38,39,40,41]. Moreover, previous studies widely reported the regenerative potential of hDPSCs in vivo, when applied to animal models of critical size bone defects [42,43,44].

Since a potential application of BGMS10 could be the treatment of bone defects caused by trauma or diseases, it would be interesting to evaluate the stem cells/scaffold interactions in order to predict the outcome of a potential implant with respect to osseointegration and osteoinduction processes.

Previous studies highlighted that the homeostasis of bone tissue is influenced by Fas ligand, a transmembrane protein that belongs to the tumor necrosis factor (TNF) family, and seems to play a key role by inducing osteoclast apoptosis. It has been widely demonstrated by several studies conducted in vitro and in vivo that hDPSCs can modulate immune/inflammatory response through different mechanisms, including Fas/FasL pathway. In particular, the activation of Fas/FasL pathway occurs following the exposure to inflammatory microenvironment which induces apoptosis in T cells [45,46]. Despite FasL expression in hDPSCs has been investigated by different research groups [38,41,46], it is still unknown whether different bioactive glass compositions can influence the expression of FasL in hDPSCs.

To this end, the aim of this study was to evaluate how and whether BGMS10 surfaces may affect hDPSCs properties, in terms of stemness, proliferation, osteogenic differentiation and immunomodulatory abilities.

## 2. Materials and Methods

### 2.1. Bioactive Glass Preparation

The preparation of BGMS10 has been extensively described elsewhere [11] and is here briefly summarised. The glass was obtained via a melt-quenching process, starting from SiO_2_, Ca_3_(PO_4_)_2_, Na_2_CO_3_, CaCO_3_, K_2_CO_3_, SrCO_3_, Mg(OH)_2_·5H_2_O (Carlo Erba Reagenti, Rodano-Milano, Italy). The raw powder reagents were melted at 1450 °C in a platinum crucible, in air. The molten glass was rapidly quenched in water to obtain a frit, subsequently left to dry at 110 °C for 12 h. Glass powders were then pressed into disks (1 cm Ø); and green bodies were heat treated at the temperature of 737 °C to obtain fully sintered samples. Both unpolished and polished disks were prepared. The polished sintered disks were prepared by lapping and polishing processes. During lapping sandpapers with different grain size were used; then, to obtain mirror surfaces diamond polishing slurry was used.

### 2.2. Bioactive Glass Characterization

First of all, the in vitro bioactivity was verified for the prepared disks. Bioactivity is often measured as the capability of the material to form a hydroxyapatite (HA) layer once in contact with physiological fluids [47,48]. The formation of a hydroxyapatite layer in vitro represents a good preliminary test often used in the literature [49]. BGMS10 showed good reactivity in simulated body fluid (SBF) for tests previously carried out on sintered samples [11] and bioactive glass granules [12]. Here, to confirm the results obtained so far, tests were repeated on polished and unpolished disks. SBF was prepared according to the Kokubo protocol [49] and stored at 37 °C. The pH was adjusted at 7.4 before use. The samples were extracted from SBF at fixed time points, i.e., 1, 7 and 14 days after immersion. The microstructure of the sample before and after SBF and the eventual hydroxyapatite layer formation were observed using an environmental scanning electron microscope, ESEM (ESEM Quanta 200-FEI Company, Eindhoven, The Netherlands), coupled with an energy-dispersive X-ray (EDS) microanalysis system (INCA, Oxford Instruments, Abingdon, UK). The microscope was operated in a low-vacuum mode with a pressure of 0.5 Torr.

Moreover, the mechanical properties of BGMS10 disks were also investigated. The micro-indentation technique was utilised to obtain both hardness and Young’s modulus of samples, using a depth-sensing instrumentation. Measurements were performed using Open-Platform equipment (CSM Instruments, Peseux, Switzerland), with a Vickers indenter tip. The load was set equal to 100 mN, with a load/unload rate of 200 mN/min. For each indentation, the load-penetration depth curve was automatically acquired. The Young’s modulus was deduced from the unloading part of the load-depth curve according to the Oliver and Pharr method [50]. Ten measurements were performed for each disk and ten different disks were tested and then average values obtained.

### 2.3. Human Dental Pulp Stem Cells (hDPSCs) Isolation and Immune Selection

This study was carried out in compliance with the recommendations of Comitato Etico Provinciale–Azienda Ospedaliero-Universitaria di Modena (Modena, Italy), which provided the approval of the protocol (ref. number 3299/CE; 5 September 2017). Human dental pulp was obtained, after routine dental extraction, from third molars of adult subjects (n = 3; 30–35 years) who gave their written informed consent according to the Declaration of Helsinki. Cells isolation from dental pulp was carried out as previously described [39]. Briefly, dental pulp was harvested from the teeth and enzymatically digested by means of 3 mg/mL type I collagenase and 4 mg/mL dispase in α-MEM (alpha modification of minimal essential medium). A cell suspension was obtained by filtering pulp onto 100 µm Falcon Cell Strainers, then cell suspension was plated in 25 cm^2^ culture flasks and expanded in standard culture medium [α-MEM supplemented with 10% heat-inactivated foetal bovine serum (FBS), 2 mM L-glutamine, 100 U/mL penicillin, 100 µg/mL streptomycin] at 37 °C and 5% CO_2_. Following cell expansion, hDPSCs were immune-selected by magnetic activated cell sorting (MACS), using MACS^®^ separation kit according to manufacturer’s instructions. Two separate sorting were carried out by using specific antibodies against STRO-1 and c-Kit. The mouse IgM anti-STRO-1 and rabbit IgG anti-c-Kit primary antibodies (Santa Cruz, Dallas, TX, USA) were detected by the following magnetically labelled secondary antibodies: anti-mouse IgM and anti-rabbit IgG (Miltenyi Biotec, Bergisch Gladbach, Germany). For each selection approximately 5 × 10^6^ cells were used. Firstly, heterogeneous pulp cell suspension was sorted by anti-STRO-1 antibody. STRO-1^+^ cells were then expanded and subsequently sorted by using anti-c-Kit antibody to obtain a STRO-1^+^/c-Kit^+^ hDPSCs population, that represents a purer stem cells population.

All the experiments were performed using STRO-1^+^/c-Kit^+^ hDPSCs.

### 2.4. Cell Morphology and Proliferation

Undifferentiated STRO-1^+^/c-Kit^+^ hDPSCs at passage 1 were seeded at a density of 2.5 × 10^3^ cells/cm^2^ on BGMS10 disks in 12-multiwell culture plates and cultured in standard conditions and maintained in an expansion medium (α-MEM, 10% FBS, 1% L-glutamine, 1% penicillin and streptomycin, all from Sigma-Aldrich, Saint Louis, MO, USA). For each experimental time point (24 h, 4 and 7 days), 6 BGMS10 disks were used and cells were fixed in 4% paraformaldehyde in phosphate-buffered saline (PBS) for 15 min without dissociating them from the BGMS10 disks. The cells were subsequently permeabilized with 0.1% Triton X-100 in PBS for 5 min, stained with AlexaFluor546-conjugated Phalloidin (Abcam, Cambridge, UK) and rinsed with PBS 1%. Nuclei were stained with 1 μg/mL 4′,6-diamidino-2-phenylindole (DAPI) in PBS 1%. DABCO (Sigma Aldrich, Saint Louis, MO, USA) was used as an anti-fading mounting medium. Cell proliferation and morphology were assessed by using confocal microscopy (Nikon A1 confocal laser scanning microscope), as formerly described by Conserva et al. [51]. Cell proliferation was evaluated by counting the DAPI-stained nuclei on 10 randomly selected fields measuring 2.85 × 10^5^ μm^2^ for each disk by an individual blinded to the experimental details.

### 2.5. Evaluation of Stemness Markers in hDPSCs Cultured on BGMS10 Disks

After 24 h and 4 days of culture on each disk, respectively, cells were fixed in 4% paraformaldehyde in PBS for 15 min and then processed as previously described [39]. The following primary antibodies were diluted 1:50 in bovine serum albumin 1% (BSA): mouse IgM anti-STRO-1 and rabbit IgG anti-c-Kit (Santa Cruz, Dallas, TX, USA). Secondary antibodies (goat anti-mouse IgM AlexaFluor488, goat anti-rabbit AlexaFluor546) were diluted 1:200 in 1% BSA (Thermo Fisher Scientific, Waltham, MA, USA). Nuclei were stained with 1 μg/mL DAPI in 1% PBS for 5 min, then disks were mounted with 1,4-diazabicyclo [2.2.2] octane solution (DABCO, Sigma Aldrich, Saint Louis, MO, USA) anti-fading medium. The multi-labelling immunofluorescence experiments were performed avoiding cross-reactions between primary and secondary antibodies. Confocal imaging was done with a Nikon A1 confocal laser scanning microscope. Fiji ImageJ software (NIH, Bethesda, MD, USA) was used in order to process confocal serial sections and to obtain 3-dimensional projections, while image rendering was performed by Adobe Photoshop Software (Photoshop 7.0, Adobe, San Jose, CA, USA) [52].

### 2.6. Evaluation of FasL Expression in hDPSCs Cultured on BGMS10 Disks

In order to investigate the maintenance of immunomodulatory properties of hDPSCs cultured on BGMS10 the expression of FasL was investigated in cells after 24 h and 4 days of culture. Rabbit anti-FasL (Santa Cruz, Dallas, TX, USA) primary antibody was used at dilution of 1:100 and then revealed by a goat anti-rabbit AlexaFluor488 secondary antibody (Thermo Fisher Scientific, Waltham, MA, USA), diluted 1:200. Nuclei were stained with 1 μg/mL DAPI in PBS. Confocal imaging was performed as described above.

### 2.7. Osteogenic Induction

In order to evaluate the ability of the BGMS10 surfaces to influence osteogenic differentiation, cells were seeded at approximately 2.5 × 10^3^ cells/cm^2^ on these disks. After one week of culture, the standard culture medium was replaced with the osteogenic medium (α-MEM, 10% FBS, 2 mM L-glutamine, 100 U/mL penicillin, 100 mg/mL di streptomycin, 100 nM dexamethasone, 10 mM di β-glycerophosphate, all from Sigma-Aldrich, St. Louis, MO, USA). After 3 weeks of induction, the expression of typical differentiation markers, such as RUNX2, osterix (Osx), osteopontin (OPN) and osteocalcin (OCN), was investigated by immunofluorescence analyses as described above. The following primary antibodies were used, at a 1:100 dilution: rabbit anti-RUNX2, mouse anti-OPN, mouse anti-OCN (Abcam, Cambridge, UK) and rabbit anti-Osx (Gene-Tex, San Antonio, TX, USA).

### 2.8. Statistical Analysis

All the experimental evaluations were performed in triplicate. Data were expressed as mean ± standard deviation (SD). Differences among three experimental samples were analyzed by analysis of variance (ANOVA) followed by Tukey’s post hoc test (GraphPad Prism Software version 5 Inc., San Diego, CA, USA). In any case, significance was set at *p* < 0.05.

## 3. Results and Discussion

### 3.1. Bioactive Glass Disks Characterisation

**Figure 1:**
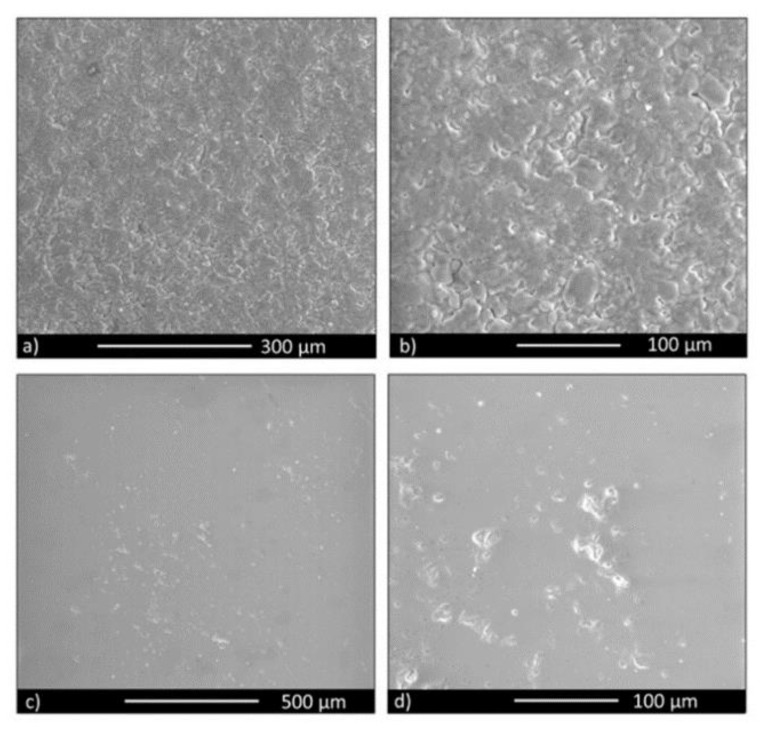
Scanning electron microscopy (SEM) observation of the BGMS10 disk surface: unpolished—low magnification (**a**); unpolished—high magnification (**b**); polished—low magnification (**c**); polished—high magnification (**d**).

Compact and fully sintered samples were obtained. In Figure 1, images for both unpolished and polished disks are reported. The scanning electron microscope (SEM) images confirm the high degree of compaction achieved during the thermal cycle. In fact, even if BGMS10 has an ultra-high crystallisation temperature (932 °C) as previously reported [11], it is possible to sinter it at relatively low temperatures, i.e., 737 °C. It is worth noting that 45S5 is usually sintered at higher temperatures and this results in extensive crystallization phenomena. Crystallization phenomena can reduce bioactivity [8,53]. Therefore, crystallization temperature is a critical issue for producing amorphous bioactive glasses [54]; a bioactive glass which undergoes partial or complete crystallization could provoke failure in the structure (i.e., scaffolds, coatings) [55]. Additionally, although it is not of pivotal importance to produce an amorphous material to have high bioactivity (which is also showed by glass-ceramics materials), the effort of research was focused on obtaining bioactive glasses that can be easily sintered without crystallization phenomena [56,57,58].

BGMS10 is characterized by a large processing window and it can be sintered without any crystallization phenomena [11,12]. This is of crucial importance, since crystallization inhibits or at least reduces bioactivity [8,59], as mentioned.

**Figure 2:**
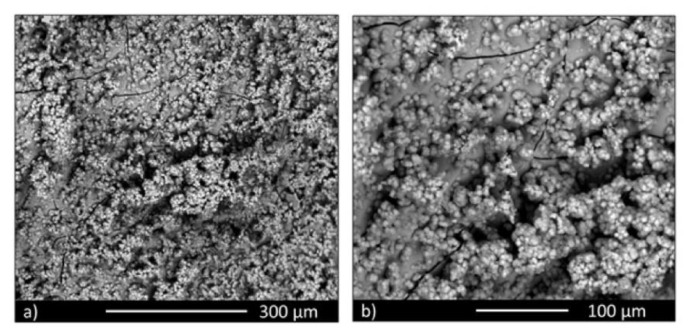
Simulated body fluid (SBF) tests: HA deposition on BGMS10 disks after 14 days of immersion (**a**) and higher magnification detail (**b**).

In vitro bioactivity was confirmed by SBF testing of disks. SBF is an acellular solution with ion concentration analogous to that of human blood plasma. As can be observed in Figure 2, an abundant hydroxyapatite layer completely covered the disk’s surface. The EDS analysis confirmed that the superficial layer had a Ca/P stoichiometric value analogous to that of hydroxyapatite (Ca/P value ≅ 1.67). Such precipitates of hydroxyapatite are similar to the biological apatite, which is the mineral component of bone. The HA (precipitated) layer is able to enhance the attachment of implant materials to bone, and to promote new bone growth. This property is known as osteoconductivity. It is likely that the response of BGMS10 is favorable also because of the presence of ions such as strontium and magnesium, whose biological relevance has been confirmed in the literature.

The mechanical properties of BGMS10 sintered disks were investigated by means of the micro-indentation technique. The Vickers hardness of sintered BGMS10 was 731.5 ± 48.0 HV (Table 1). It can be hypothesized that the substitution of some CaO with MgO and SrO had an effect on the increase of hardness of the sintered bioactive glass. In fact, the comparison of these results with those obtained in [60] for the sintered BG_Ca-Mix (564 ± 47 HV) corroborates this hypothesis. Therefore, the increase of hardness could be ascribed to the strength of metal-oxygen bond which varies as MgO > CaO > SrO > BaO [61].

On the other hand, the elastic modulus of BGMS10 was 54.51 ± 3.0 (Table 1). Such a value is higher compared to that of 45S5 Bioglass^®^ and lower than the BG_Ca-Mix (35 GPa the former and between 65 GPa and 122 GPa the latter [62,63]). However, even though the elastic modulus of BGMS10 was lower, BGMS10 disks were well sintered (as reported in Figure 1).

### 3.2. Stem Cells Morphology and Proliferation on BGMS10 Disks

**Figure 3:**
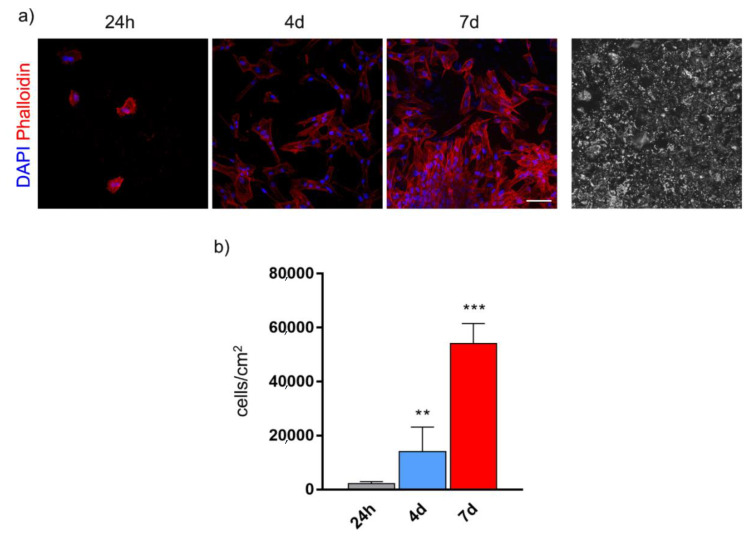
Cell morphology (**a**) and proliferation (**b**) of human dental pulp stem cells (hDPSCs) cultured on BGMS10 surfaces at different time points. Immunofluorescence analysis shows representative images of hDPSCs at 24 h, 4 days and 7 days. On the right, BGMS10 surface is shown. ** *p* < 0.01 vs. 24 h, *** *p* < 0.001 7 days vs. 4 days and vs. 24 h. Scale bar: 50 μm.

In order to evaluate hDPSCs morphology, immunofluorescence analysis was performed on cells stained with phalloidin and DAPI at three different time points: 24 h, 4 days and 7 days. As shown in Figure 3a, as early as at 24 h of culture cells showed a round-shaped appearance which, after 4 and 7 days of culture, was shifted to the typical fibroblast morphology. These data suggest that BGMS10 composition proves to be favorable to cell attachment and to the maintenance of cell morphology.

With regard to cell proliferation, as shown in the histograms (Figure 3b), data highlighted a statistically significant increase in cell number after 4 and 7 days of culture (** *p* < 0.01 4 days vs. 24 h, *** *p* < 0.001 7 days vs. 24 h and 7 days vs. 4 days), demonstrating that BGMS10 is suitable for hDPSCs growth through extended culture times.

### 3.3. Effect of BGMS10 on hDPSCs on Stemness and Immunomodulatory Markers

**Figure 4:**
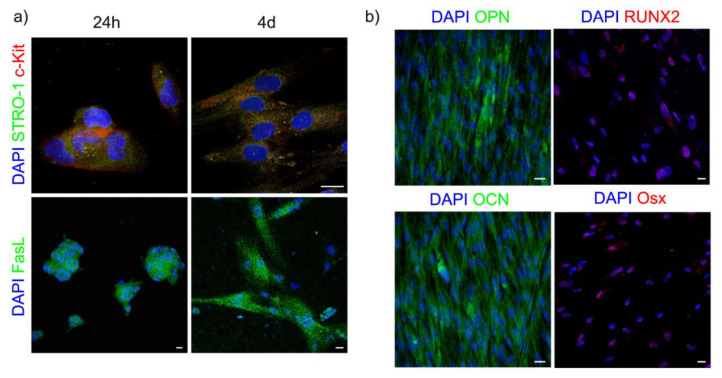
(**a**) Evaluation of stemness markers and FasL expression in hDPSCs cultured on BGMS10 at 24 h and 4d. (**b**) Osteogenic differentiation of hDPSCs after 21 days of induction on BGMS10 surfaces. Scale bar: 10 μm.

hDPSCs were immune-selected against STRO-1 and c-Kit and seeded on BGMS10 disks. In order to evaluate the maintenance of their stemness properties, after 24 h and 4 days of culture, immunofluorescence analysis was performed. To this purpose, the expression of STRO-1 and c-Kit, two typical stemness markers, was investigated. As shown in Figure 4a, BGMS10 did not affect the expression of the stemness markers STRO-1 and c-Kit, proving that the stemness properties of hDPSCs, including the ability to proliferate and differentiate, were preserved when cultured on these surfaces.

In parallel, as shown in Figure 4a, confocal immunofluorescence microscopy also revealed that hDPSCs cultured for 24 h and 4 days on BGMS10 surfaces expressed FasL, a key marker related to stem cells immunomodulatory abilities. As well known from the literature, mesenchymal stem cells and hDPSCs as well are characterized by immunomodulatory properties that can be exerted through different mechanisms, including the Fas/FasL pathway and soluble factors [41,45,46]. It is noteworthy that when cultured on BGMS10, hDPSCs not only maintained the expression of stemness markers but also were still positively labeled against FasL. To this end, when evaluating the cells/material interactions it is primary to keep in consideration how to promote the osseointegration of the implant. As a matter of fact, a surface capable to maintain the immunomodulatory properties of stem cells would be suitable for the modulation of inflammatory processes often occurring post-implantation [51].

### 3.4. Osteogenic Induction

After 3 weeks of osteogenic induction, the expression of the osteogenic markers RUNX2, OPN, OSX and OCN in hDPSCs seeded on BGMS10 surfaces were evaluated by fluorescence confocal microscopy analysis. At the end of osteogenic induction, almost all hDPSCs seeded on BGMS10 disks showed the expression of RUNX2 and Osx, bone-related transcription factors, and at the same time were positively labeled against OPN and OCN, revealing an ongoing deposition of mineralized extracellular matrix (Figure 4b). Based on this evidence, it can be argued that BGMS10 composition is able to trigger the osteogenic commitment of hDPSCs after shorter times of induction when compared to previous findings reporting the ability to reach the osteogenic differentiation at later times under standard culture conditions [64].

## 4. Conclusions

As mentioned, one of the main advantages of bioactive glasses is that their composition can be tailored, also with the addition of therapeutic ions, to induce a specific response in the host or to introduce functionalities. Therefore, bioactive glasses can be advantageously employed for their favourable combination of properties.

Taking advantage of the use of hDPSCs, which constitute a stem cell population with peculiar properties related to their embryological origin and immunomodulation potential, our study provided evidence that BGMS10 surfaces are able to preserve such stemness properties and to promote the deposition of bone matrix-related proteins, due to the osteoconductivity of the surface. Interestingly, the BGMS10 surface did not alter the immunomodulatory abilities of hDPSCs, which are primary for the success of the implant and to reach osseointegration.

Therefore, our study demonstrates that BGMS10 can represent an adequate tool for future potential application in orthopedic and dental implants.

## Figures and Tables

**Table 1:** Mechanical properties of BGMS10 disks, as determined by depth-sensing micro-indentation: elastic modulus and hardness.

Young’s Modulus [GPa]	Hardness [Vickers]
54.51 ± 3.0	731.5 ± 48.0

## References

[1] Griffith L.G. (2000). Polymeric biomaterials. Acta Mater..

[2] Boccaccini A.R., Gough J. (2007). Tissue Engineering Using Ceramics and Polymers.

[3] Baino F., Novajra G., Miguez-Pacheco V., Boccaccini A.R., Vitale-Brovarone C. (2016). Bioactive glasses: Special applications outside the skeletal system. J. Non-Cryst. Solids.

[4] Jones J.R., Brauer D.S., Hupa L., Greenspan D.C. (2016). Bioglass and Bioactive Glasses and Their Impact on Healthcare. Int. J. Appl. Glass Sci..

[5] Hench L.L. (1998). Biomaterials: A forecast for the future. Biomaterials.

[6] Pirhonen E. (2006). Fibres and Composites for Potential Biomaterials Applications. Ph.D. Thesis.

[7] Hench L.L., Polak J.M. (2002). Third-Generation Biomedical Materials. Science.

[8] Jones J.R. (2015). Reprint of: Review of bioactive glass: From Hench to hybrids. Acta Biomater..

[9] Kaur G., Pandey O.P., Singh K., Homa D., Scott B., Pickrell G. (2014). A review of bioactive glasses: Their structure, properties, fabrication, and apatite formation. J. Biomed. Mater. Res. Part A.

[10] Liang W., Rüssel C., Day D.E., Völksch G. (2006). Bioactive comparison of a borate, phosphate and silicate glass. J. Mater. Res..

[11] Bellucci D., Cannillo V. (2018). A novel bioactive glass containing strontium and magnesium with ultra-high crystallization temperature. Mater. Lett..

[12] Bellucci D., Veronesi E., Strusi V., Petrachi T., Murgia A., Mastrolia I., Dominici M., Cannillo V. (2019). Human Mesenchymal Stem Cell Combined with a New Strontium-Enriched Bioactive Glass: An ex-vivo Model for Bone Regeneration. Materials.

[13] Bellucci D., Veronesi E., Dominici M., Cannillo V. (2020). On the in Vitro Biocompatibility Testing of Bioactive Glasses. Materials.

[14] Yang F., Yang D., Tu J., Zheng Q., Cai L., Wang L. (2011). Strontium enhances osteogenic differentiation of mesenchymal stem cells and in vivo bone formation by activating Wnt/catenin signaling. Stem Cells.

[15] Massera J., Kokkari A., Närhi T., Hupa L. (2015). The influence of SrO and CaO in silicate and phosphate bioactive glasses on human gingival fibroblasts. J. Mater. Sci. Mater. Med..

[16] Cacciotti I. (2017). Bivalent cationic ions doped bioactive glasses: The influence of magnesium, zinc, strontium and copper on the physical and biological properties. J. Mater. Sci..

[17] Rabiee S.M., Nazparvar N., Azizian M., Vashaee D., Tayebi L. (2015). Effect of ion substitution on properties of bioactive glasses: A review. Ceram. Int..

[18] Ma J., Chen C.Z., Wang D.G., Jiao Y., Shi J.Z. (2010). Effect of magnesia on the degradability and bioactivity of sol-gel derived SiO_2_-CaO-MgO-P_2_O_5_ system glasses. Colloids Surf. B Biointerfaces.

[19] Aydin H. (2013). Magnesium supplementation and bone. Magnes. Hum. Health Dis..

[20] Greenspan D.C. (2016). Glass and Medicine: The Larry Hench Story. Int. J. Appl. Glass Sci..

[21] Bellucci D., Salvatori R., Giannatiempo J., Anesi A., Bortolini S., Cannillo V. (2019). A New Bioactive Glass/Collagen Hybrid Composite for Applications in Dentistry. Materials.

[22] Bellucci D., Salvatori R., Anesi A., Chiarini L., Cannillo V. (2019). SBF assays, direct and indirect cell culture tests to evaluate the biological performance of bioglasses and bioglass-based composites: Three paradigmatic cases. Mater. Sci. Eng. C.

[23] Elsayed H., Rincon Romero A., Bellucci D., Cannillo V., Bernardo E. (2019). Advanced Open-Celled Structures from Low-Temperature Sintering of a Crystallization-Resistant Bioactive Glass. Materials.

[24] Sergi R., Bellucci D., Salvatori R., Cannillo V. (2020). Chitosan-Based Bioactive Glass Gauze: Microstructural Properties, In Vitro Bioactivity, and Biological Tests. Materials.

[25] Sergi R., Cannillo V., Boccaccini A.R., Liverani L. (2020). Incorporation of Bioactive Glasses Containing Mg, Sr, and Zn in Electrospun PCL Fibers by Using Benign Solvents. Appl. Sci..

[26] Sergi R., Bellucci D., Salvatori R., Maisetta G., Batoni G., Cannillo V. (2019). Zinc containing bioactive glasses with ultra-high crystallization temperature, good biological performance and antibacterial effects. Mater. Sci. Eng. C.

[27] Zhang Y., Cui X., Zhao S., Wang H., Rahaman M.N., Liu Z., Huang W., Zhang C. (2015). Evaluation of injectable strontium-containing borate bioactive glass cement with enhanced osteogenic capacity in a critical-sized rabbit femoral condyle defect model. ACS Appl. Mater. Interfaces.

[28] Wang X., Li X., Ito A., Sogo Y. (2011). Synthesis and characterization of hierarchically macroporous and mesoporous CaO-MO-SiO 2-P 2O 5 (M = Mg, Zn, Sr) bioactive glass scaffolds. Acta Biomater..

[29] Zhou J., Wang H., Zhao S., Zhou N., Li L., Huang W., Wang D., Zhang C. (2016). In vivo and in vitro studies of borate based glass micro-fibers for dermal repairing. Mater. Sci. Eng. C.

[30] Rakhshaei R., Namazi H. (2017). A potential bioactive wound dressing based on carboxymethyl cellulose/ZnO impregnated MCM-41 nanocomposite hydrogel. Mater. Sci. Eng. C.

[31] Liu J., Rawlinson S.C.F., Hill R.G., Fortune F. (2016). Strontium-substituted bioactive glasses in vitro osteogenic and antibacterial effects. Dent. Mater..

[32] Fernandes J.S., Gentile P., Pires R.A., Reis R.L., Hatton P.V. (2017). Multifunctional bioactive glass and glass-ceramic biomaterials with antibacterial properties for repair and regeneration of bone tissue. Acta Biomater..

[33] Baino F., Verné E. (2017). Glass-based coatings on biomedical implants: A state-of-the-art review. Biomed. Glasses.

[34] Henao J., Poblano-salas C., Monsalve M., Corona-castuera J. (2019). Bio-active glass coatings manufactured by thermal spray: A status report. Integr. Med. Res..

[35] Gronthos S., Brahim J., Li W., Fisher L.W., Cherman N., Boyde A., DenBesten P., Robey P.G., Shi S. (2002). Stem Cell Properties of Human Dental Pulp Stem Cells. J. Dent. Res..

[36] Laino G., Carinci F., Graziano A., d’Aquino R., Lanza V., De Rosa A., Gombos F., Caruso F., Guida L., Rullo R. (2006). In Vitro Bone Production Using Stem Cells Derived from Human Dental Pulp. J. Craniofac. Surg..

[37] Waddington R.J., Youde S.J., Lee C.P., Sloan A.J. (2009). Isolation of Distinct Progenitor Stem Cell Populations from Dental Pulp. CTO.

[38] Kim B.-C., Bae H., Kwon I.-K., Lee E.-J., Park J.-H., Khademhosseini A., Hwang Y.-S. (2012). Osteoblastic/cementoblastic and neural differentiation of dental stem cells and their applications to tissue engineering and regenerative medicine. Tissue Eng. Part B Rev..

[39] Zordani A., Pisciotta A., Bertoni L., Bertani G., Vallarola A., Giuliani D., Puliatti S., Mecugni D., Bianchi G., de Pol A. (2019). Regenerative potential of human dental pulp stem cells in the treatment of stress urinary incontinence: In vitro and in vivo study. Cell Prolif..

[40] Kolar M.K., Itte V.N., Kingham P.J., Novikov L.N., Wiberg M., Kelk P. (2017). The neurotrophic effects of different human dental mesenchymal stem cells. Sci. Rep..

[41] Pisciotta A., Bertani G., Bertoni L., Di Tinco R., De Biasi S., Vallarola A., Pignatti E., Tupler R., Salvarani C., de Pol A. (2020). Modulation of Cell Death and Promotion of Chondrogenic Differentiation by Fas/FasL in Human Dental Pulp Stem Cells (hDPSCs). Front. Cell Dev. Biol..

[42] D’Aquino R., De Rosa A., Lanza V., Tirino V., Laino L., Graziano A., Desiderio V., Laino G., Papaccio G. (2009). Human mandible bone defect repair by the grafting of dental pulp stem/progenitor cells and collagen sponge biocomplexes. Eur. Cell Mater..

[43] Otaki S., Ueshima S., Shiraishi K., Sugiyama K., Hamada S., Yorimoto M., Matsuo O. (2007). Mesenchymal progenitor cells in adult human dental pulp and their ability to form bone when transplanted into immunocompromised mice. Cell Biol. Int..

[44] De Mendonça Costa A., Bueno D.F., Martins M.T., Kerkis I., Kerkis A., Fanganiello R.D., Cerruti H., Alonso N., Passos-Bueno M.R. (2008). Reconstruction of large cranial defects in non-immunosuppressed experimental design with human dental pulp stem cells. J. Craniofac. Surg..

[45] Pierdomenico L., Bonsi L., Calvitti M., Rondelli D., Arpinati M., Chirumbolo G., Becchetti E., Marchionni C., Alviano F., Fossati V. (2005). Multipotent Mesenchymal Stem Cells with Immunosuppressive Activity Can Be Easily Isolated from Dental Pulp. Transplantation.

[46] Zhao Y., Wang L., Jin Y., Shi S. (2012). Fas Ligand Regulates the Immunomodulatory Properties of Dental Pulp Stem Cells. J. Dent. Res..

[47] Verné E., Bretcanu O., Balagna C., Bianchi C.L., Cannas M., Gatti S., Vitale-Brovarone C. (2009). Early stage reactivity and in vitro behavior of silica-based bioactive glasses and glass-ceramics. J. Mater. Sci. Mater. Med..

[48] Zadpoor A.A. (2014). Relationship between in vitro apatite-forming ability measured using simulated body fluid and in vivo bioactivity of biomaterials. Mater. Sci. Eng. C.

[49] Kokubo T., Takadama H. (2006). How useful is SBF in predicting in vivo bone bioactivity?. Biomaterials.

[50] Oliver W.C., Pharr G.M. (1992). An improved technique for determining hardness and elastic modulus using load and displacement sensing indentation experiments. J. Mater. Res..

[51] Conserva E., Pisciotta A., Borghi F., Nasi M., Pecorini S., Bertoni L., de Pol A., Consolo U., Carnevale G. (2019). Titanium Surface Properties Influence the Biological Activity and FasL Expression of Craniofacial Stromal Cells. Stem Cells Int..

[52] Carnevale G., Carpino G., Cardinale V., Pisciotta A., Riccio M., Bertoni L., Gibellini L., De Biasi S., Nevi L., Costantini D. (2017). Activation of Fas/FasL pathway and the role of c-FLIP in primary culture of human cholangiocarcinoma cells. Sci. Rep..

[53] Mancuso E., Bretcanu O.A., Marshall M., Birch M.A., McCaskie A.W., Dalgarno K.W. (2017). Novel bioglasses for bone tissue repair and regeneration: Effect of glass design on sintering ability, ion release and biocompatibility. Mater. Des..

[54] Arstila H., Vedel E., Hupa L., Hupa M. (2007). Factors affecting crystallization of bioactive glasses. J. Eur. Ceram. Soc..

[55] Filho O.P., La Torre G.P., Hench L.L. (1996). Effect of crystallization on apatite-layer formation of bioactive glass 45S5. J. Biomed. Mater. Res..

[56] Weinberg M.C. (1994). Glass-forming ability and glass stability in simple systems. J. Non-Cryst. Solids.

[57] Kapoor S., Goel A., Pascual M.J., Ferreira J.M. (2016). Alkali-free bioactive diopside-tricalcium phosphate glass-ceramics for scaffold fabrication: Sintering and crystallization behaviours. J. Non-Cryst. Solids.

[58] Lara C., Pascual M.J., Durán A. (2004). Glass-forming ability, sinterability and thermal properties in the systems RO-BaO-SiO2 (R = Mg, Zn). J. Non-Cryst. Solids.

[59] Nychka J.A., Mazur S.L.R., Kashyap S., Li D., Yang F. (2009). Dissolution of bioactive glasses: The effects of crystallinity coupled with stress. JOM.

[60] Bellucci D., Sola A., Cannillo V. (2012). Low temperature sintering of innovative bioactive glasses. J. Am. Ceram. Soc..

[61] Kaur G., Pickrell G., Kumar V., Pandey O.P., Singh K., Arya S.K. (2015). Mechanical, dielectric and optical assessment of glass composites prepared using milling technique. Bull. Mater. Sci..

[62] Desogus L., Cuccu A., Montinaro S., Orrù R., Cao G., Bellucci D., Sola A., Cannillo V. (2015). Classical Bioglass^®^ and innovative CaO-rich bioglass powders processed by Spark Plasma Sintering: A comparative study. J. Eur. Ceram. Soc..

[63] Bellucci D., Sola A., Salvatori R., Anesi A., Chiarini L., Cannillo V. (2017). Role of magnesium oxide and strontium oxide as modifiers in silicate-based bioactive glasses: Effects on thermal behaviour, mechanical properties and in-vitro bioactivity. Mater. Sci. Eng. C.

[64] Laino G., Graziano A., d’Aquino R., Pirozzi G., Lanza V., Valiante S., De Rosa A., Naro F., Vivarelli E., Papaccio G. (2006). An approachable human adult stem cell source for hard-tissue engineering. J. Cell. Physiol..

